# Bioengineering the Future: Tomato Peel Cutin as a Resource for Medical Textiles

**DOI:** 10.3390/polym17060810

**Published:** 2025-03-19

**Authors:** Gianni Pecorini, Martina Tamburriello, Erika Maria Tottoli, Ida Genta, Bice Conti, Maria Nelly Garcia Gonzalez, Rita Nasti, Rossella Dorati

**Affiliations:** 1 Department of Drug Sciences, University of Pavia, Viale Taramelli 12, 27100 Pavia, Italy; 2 Environmental and Energy Systems Studies, Department of Technology and Society, Lund University, P.O. Box 118, SE-221 00 Lund, Sweden; 3 Department of Environmental Science and Policy (ESP), Università degli Studi di Milano, Via Celoria 2, 20133 Milano, Italy

**Keywords:** agro-waste, cutin, medical textile, electrospinning

## Abstract

The exponential increase in medical waste production has increased the difficulty of waste management, resulting in higher medical waste dispersion into the environment. By employing a circular economy approach, it is possible to develop new materials by waste valorization. The employment of biodegradable and renewable agro-food, waste-derived materials may reduce the environmental impact caused by the dispersion of medical waste. In this work, tomato peel recovered cutin was blended with poly(L-lactide-co-ε-caprolactone) (PLAPCL) to develop new textiles for medical application through electrospinning. The textile fabrication process was studied by varying Cut content in the starting suspensions and by optimizing fabrication parameters. Devices with dense and porous structures were developed, and their morphological, thermal, and physical–chemical properties were evaluated through scanning electron microscopy, differential scanning calorimetry, thermogravimetric analysis, and Fourier transformed infrared spectroscopy. Textile material stability to γ-irradiation was evaluated through gel permeation chromatography, while its wettability, mechanical properties, and biocompatibility were analyzed through contact angle measurement, tensile test, and MTT assay, respectively. The LCA methodology was used to evaluate the environmental impact of textile production, with a specific focus on greenhouse gas (GHG) emissions. The main results demonstrated the suitability of PLAPCL–cutin blends to be processed through electrospinning and the obtained textile’s suitability to be used to develop surgical face masks or patches for wound healing.

## 1. Introduction

Globally, medical waste is considered to be the most hazardous, after radioactive waste. Such waste is generated by medical facilities, research centers, and morgues, and if mismanaged it can affect human health and the environment [1]. The management of healthcare waste has become more relevant, especially after the 2019 pandemic of the Coronavirus Disease (COVID-19). The average amount of medical waste from hospitals, which was only 0.5 kg/bed per day before the pandemic, increased to 3.4 kg/bed per day during COVID-19 [2]. Specifically, the Italian Institute for Environmental Protection and Research (ISPRA) report that, in Italy, medical waste generation in 2021 increased by 13.4% over the previous year, exceeding 265 thousand tons. Of the total, 239 thousand tons were hazardous medical waste: waste with infectious risk [3]. In addition, the exponential increase in waste material, which was occurring in an already precarious situation, has further aggravated waste management operations: due to the increase in the number of patients to be treated, the ability of health care workers to classify waste materials has diminished, thus affecting their safe management [4]. The challenge of increasing and managing waste, argues the World Health Organization (WHO), has provided an opportunity for practical and sustainable management of health care waste. These solutions include education on the responsible use of personal protective equipment (PPE); the reduction of product packaging; and the use of renewable, biobased, or recyclable materials [4]. To solve these problems, current research has approached a sustainable model, which has as its focus the concept of biobased materials, grounded in the circular economy [5]. The foundation on which the circular economy is based reverses the traditional linear economy model. In fact, the circular economy promotes reuse, repair, and recycling of resources, thus developing a “closed system” that limits resource loss and extends product life [6]. Awareness has been gained that, from some agricultural waste, it is possible to obtain, through targeted steps, biopolymers and bioactive molecules that could find application in the food packaging sector, but also in the construction, manufacturing, and pharmaceutical sectors [7]. Despite having a strong impact in the field of scientific research, the upcycling of these wastes is still not a common practice, and most food companies opt for landfill disposal [8]. This is the case in the agricultural sector with tomato crops, whose annual processing of the world’s production produces 5–30% agricultural waste, destined for animal feed or landfill [9]. In line with the valorization of agri-food waste, this paper proposes the use of cutin, a second raw material derived from tomato peel, as a viable alternative to the non-biodegradable plastics still used in the biomedical field. Cutin is a biodegradable, non-toxic fatty acid polyester derived from the peel of fruits and vegetables [10], including tomatoes, where it presents a high percentage [11]. In view of its properties, several works in the literature use cutin as a biopolymer to produce antimicrobial oligomers [12] for the development of fully biobased coatings for general metal protection applications [13], or to produce wraps and films for food packaging [14,15]. Unlike the proposed applications, this paper introduces a novel approach by blending cutin with a biodegradable and biocompatible copolymer, poly(L-lactide-*co*-ε-caprolactone) (PLAPCL), to produce a biobased medical textile through the electrospinning technique. The electrospinning process allows polymeric micro/nanofibers with high surface-to-volume ratio in which low molecular weight drugs, natural substances, or nanoparticles can be incorporated and provides improved flexibility and mechanical performance [16]. The advantageous textile characteristics have enabled these nonwoven materials to find various medical applications [17]. Moreover, compared with other technologies, electrospinning techniques are more sustainable and advantageous, especially for thermally labeled active ingredients, due to the use of electricity that directly ensures drying [18]. The demand for medical textiles has grown dramatically because of the pandemic emergency [19]; in fact, health and hygiene products such as masks and surgical gowns and even wipes have found great application in hospital wards and surgical rooms [20]. However, medical textiles also find applications as implantable products, useful in suturing wounds or as blood vessel or damaged heart valve substitutes; non-implantable products such as bandages, plasters, compression garments, and surgical gauze, developed to protect, support the injured body part, and absorb exudate; and finally, as extracorporeal devices, including mechanical lungs, created to support the function of various organs [20].

Biodegradable textiles are constituted of both biobased materials (e.g., polyhydroxyalkanoates) and petroleum-based materials (e.g., polyglycolic acid and polybutylene adipate-*co*-terephthalate). To the best of the authors’ knowledge, this is the first medical textile constituted of a biobased material obtained from an agro-food waste [21]. It does not require dedicated cultivation areas or fermentation processes, and it determines a reduction of the production costs since the raw material is waste [22]. Moreover, since it is a biobased material its utilization will reduce oil depletion and reduce greenhouse gas (GHG) emissions in comparison with the utilization of petroleum-based materials such as polyglycolic acid and polybutylene adipate-*co*-terephthalate [23]. Studies on LCA for masks that are either fully or partially biobased are currently unavailable, highlighting the novelty of this study. Existing evaluations of biobased masks primarily focus on their filtration and breathability properties [24]. Environmental sustainability assessments, when conducted, are based on the production of fibers derived from biobased materials [24].

In this paper, the valorization of agricultural waste is combined with the dual goal of reducing the employment of petroleum-based materials and reusing food waste for the development of high added value biomedical textiles for application in wound healing, and as a filtering layer in face masks.

## 2. Materials and Methods

### 2.1. Materials

Cutin (Cut) was obtained following a previously patented method [25]. Briefly, supercritical carbon dioxide (sc-CO_2_) was employed for purification of cutin from tomato peel, according to the selectivity usually associated with this technique, allowing high reproducibility of the extracted product, and to growing interest in its industrial application, in particular for the isolation of natural compounds from plants and agro-waste [26]. It was depolymerized through methanolysis in an alkaline environment and further purified by precipitation in ethyl acetate (EtOAc), following the protocol outlined in a patented procedure [25]. Poly(L-lactide-*co*-ε-caprolactone) (PLAPCL) Purasorb PLC 8516 (L-lactide/caprolactone 85:15 mol/mol, M_n_ 112,000 Da) was purchased from Corbion (Amsterdam, The Netherlands) and used without further purification. Dichloromethane (DCM), ethanol (EtOH), and N,N-dimethylformamide (DMF) were purchased from Carlo Erba and used without further purification.

### 2.2. Solutions Preparation

For the preparation of PLAPCL solutions, the selected amount of polymer was dissolved in DCM in a sealed glass vial under magnetic stirring. After polymer compete dissolution, EtOH and DMF were added. The final solvent composition was 94.1% *v*/*v* DCM, 3.3% *v*/*v* EtOH, and 2.6% *v*/*v* of DMF. The obtained PLAPCL solution was left under magnetic stirring overnight.

For the preparation of Cut suspensions, Cut was poured into a glass vial, together with EtOH, and it was heated at 80 °C under magnetic stirring for 1 h to obtain a stable suspension. DCM was poured in a different glass vial, together with PLAPCL, and after complete dissolution it was transferred and mixed with the Cut solution. After homogenization, the corresponding amount of DMF was added and the suspension was left under magnetic stirring overnight before use. In Table 1 are reported the optimized concentrations of PLAPCL and Cut, and the composition of the solvent of the PLAPCL and PLAPCL–Cut solutions/suspensions.

### 2.3. Solutions Characterization

#### 2.3.1. Rheological Measurements

Rheological measurements were carried out, employing a Rotational Rheometer Malvern Kinexus Pro+ (Malvern, Alphatest, Milan, Italy) equipped with a CP4/40 flat cone geometry (CP4/40 S0172 SS 40 mm diameter, 1° cone angle) at 32 °C. The amplitude sweep tests were performed at 32 °C, at a constant frequency of 1 Hz, and shear strain ranged from 10^−3^ to 10^2^%. Shear rate ramp analysis was performed from 10^−3^ to 10 s^−1^ at 32 °C. Three replicates of each solution were analyzed, and curves were built using viscosity data obtained as the average of the three measurements.

#### 2.3.2. Measurement of Surface Tension

The surface tension of the developed solutions was measured, employing a DuNouy Tensiometer equipped with a 6.28 cm diameter ring. Deionized water was selected as a control. Three measurements were carried out for each solution, and the results were reported as mean ± standard deviation.

### 2.4. Fabrication of PLAPCL–Cut Fibers Through Electrospinning

The processing of the solutions was carried out by employing Nanon-01 Electrospinning (MECC Nanofiber, Fukuoka, Japan). The solutions were transferred to a plastic syringe equipped with a metallic needle (22 Gau inner diameter), and the syringe was placed on the instrument syringe pump set on 0.8 mL/h flow rate and which was able to translate along the x-axis. The syringe translation distance was set at 8 cm at 1 cm/s translation speed. The distance between the needle tip and the collector, constituted by a metallic plate, was 15 cm. A 25 kV voltage was applied at the needle tip, while the collector was kept at 0 kV. Working temperature and humidity were maintained between 30 and 32 °C and between 25 and 30%, respectively. Samples were stored at 4 °C. Two different typologies of samples were fabricated by employing two different collectors, both constituted by a metallic plate either with or without round pores and named Round and Plain, respectively. The Round plate specifics were reported the same by Tottoli et al. 2022 [27] and according to Patent WO2021064673A1. Briefly, the Round plate collector was constituted by a square metal plate with side lengths of 15 cm, with 1548 round pores of 1.41 mm diameter, resulting in a total pore area of 892.7 mm^2^.

### 2.5. Scanning Electron Microscopy

Samples were gold sputtered, and scanning electron microscopy (SEM) analysis was carried out on the top view of the fabricated samples by employing a Zeiss EVO MA10 (Zeiss, Oberkochen, Germany). Micrographs were obtained under high vacuum (50 Pa), employing a voltage of 20 kV. Fiber diameters were determined by analyzing 2000× micrographs, employing Image J 1.43u software. Data are reported as mean ± standard deviation calculated over 50 measurements.

### 2.6. Determination of Encapsulation Efficiency

For the determination of the encapsulation efficiency, 20 mg of PL–Cut sample were dissolved in 1 mL of THF and analyzed through GPC (see Section 2.7). Cut peak height was correlated with a calibration curve to determine its concentration in the sample. The Cut calibration curve was obtained by analyzing through GPC analysis Cut solutions at different concentrations, ranging from 1 to 6 mg/mL. The points were fitted by a linear equation, y = 2169.4x − 220.54, with a correlation coefficient of 0.9703.

Encapsulation efficiency was calculated according to Equation (1),

(1) $$\frac{C_{Cut}}{C_{Cut,th}}\times 100$$

where C_Cut_ is the Cut concentration in the sample measured through GPC analysis and C_Cut,th_ is the theoretical Cut concentration of the same sample calculated considering the amount of Cut added to the starting solution (18% *w*/*w* of PLAPCL corresponding to 16%wt. of the sample). The analysis was carried out on three different samples.

### 2.7. Gel Permeation Chromatography

Samples were dissolve in THF (20 mg/mL for encapsulation efficiency measurement, 5 mg/mL for stability studies; see Section 2.9) and analyzed using a 1260 Infinity GPC apparatus (Agilent Technologies, Santa Clara, CA, USA) equipped with three columns (Plgel 5 µm 500 Å–300 × 7.5 mm, PL aquagel-OH MIXED-H 8 µm 10 × 10^3^ Å 300 × 7.5 mm, Phenogel 5 µm 10 × 10^4^ Å), a pre-column (Plgel 5 µm 50 × 7.8 mm), and a refractive index detector. Tetrahydrofuran (THF) was employed as the mobile phase and to dissolve the samples. A flow rate of 0.8 mL/min was employed for the analysis. The calibration curve was built using polystyrene standards in the molecular weight range 4490–316,500 Da. Number average molecular weight (M_n_), average molecular weight (M_w_), and polydispersity index (PDI) were obtained from the chromatograms. Three replicates were analyzed for each sample.

### 2.8. Thermogravimetric Analysis

TGA was performed on purified Cut using a TGA400 instrument (PerkinElmer Inc., Waltham, MA, USA) in the temperature range 30–600 °C, at a heating rate of 10 °C/min, and under a nitrogen flow of 60 mL/min.

### 2.9. Differential Scanning Calorimetry

DSC analysis was performed on purified Cut under a nitrogen flow of 80 mL/min using a DSC1 instrument (Mettler Toledo, Milan, Italy) by means of one heating cycle in the range 30 to 300 °C at a heating rate of 10 °C/min. Cut melting temperature (T_m_) and melting enthalpy (ΔH_m_) were obtained from the thermograms relevant to the first heating scan. For both TGA and DSC analysis, a single replicate was analyzed.

### 2.10. γ-Sterilization

Raw polymers and textile samples were irradiated using a ^60^Co gamma radiation source (Gammatom S.r.l., Irradiation Department, Guanzate, CO, Italy) at a nominal dose of 25 kGy in the presence of air and at room temperature. To ensure sterility and prevent contamination, each sample was enclosed in gamma-compatible protective bags, which do not interfere with the irradiation process. Thermometric monitoring verified that the sample temperature remained stable and did not significantly exceed room temperature throughout the irradiation procedure.

### 2.11. Contact Angle Measurement

Contact angle measurements were carried out using a Sessile Drop DMe-211 plus (Kyowa Interface Science Co., Saitama, Japan) instrument and the FAMAS software version 5.0.30. They were measured by dropping 10 μL of water on the top surface of the samples and taking a picture 1 s after the droplet touched the sample. Analysis was carried out at room temperature.

### 2.12. Tensile Test

Mechanical characterization was carried out through a tensile test using a MARK-10 Force Gauge M5-5 instrument (Mark-10 Corporation, New York, NY, USA), and data were elaborated by employing MESUR Gauge Plus (Copiague, NY, USA) software version 2.0.5. After fabrication, samples were incubated in distilled water for 3, 6, and 20 h in a climate chamber (Body100, Pbi, Milan, Italy), kept at 37 ± 2 °C and relative humidity of 55%. Samples were cut in a standardized shape according to ISO and tests were carried out according to ASTM D882-18 [28] standard, employing a speed of testing of 1.5 mm/min.

Stress–strain curves were obtained from the software MESUR Gauge Plus version 2.0.5 (Copiague, NY, USA). The stress was calculated by dividing the measured force (F) by the area (A) of the apparent cross-section of the sample, as reported in Equation (2).

(2) Stress=F/A

The strain was defined as the ratio between the sample height variation (h − h_0_) and its initial height (h_0_), as reported in Equation (3).

(3) $$Strain=\frac{\left(h-h_{0}\right)}{h_{0}}\times 100$$

Elastic modulus was calculated as the slope of the initial linear region of the stress–strain curve according to Equation (4), while tensile strength was determined as the maximum stress measured by the instrument, and elongation at break was calculated as the strain at the specimen rupture point.

(4) $$Elastic\;modulus=\frac{{Stress}_{2}-{Stress}_{1}}{{Strain}_{2}-{Strain}_{1}}\times 100$$

### 2.13. Biological Characterization

#### 2.13.1. Cell Culture

Fibroblasts were derived from a primary culture (NHDF, Dermal Fibroblast Adult, amp 500 kCells, Lonza, Milan, Italy). The cells were resuspended in DMEM completed with 10% *v*/*v* of Fetal Bovine Serum (FBS), 1% *v*/*v* of antibiotic mixture (100 µg/mL penicillin and 100 µg/mL streptomycin), 1% *v*/*v* Glutamine, and 2% Sodium Pyruvate. The fibroblasts were suspended in DMEM and centrifuged for 5 min at 1500 rpm (Sorvall TC & Centrifuge, Thermofisher, Waltham, MA, USA); the pellet was resuspended in fresh media and incubated at 37 °C and 5% CO_2_. During the proliferation, the morphology and growth of cells were evaluated daily with a reverse optic microscope (LEICA DM 13000B, Leica Microsystems, Milan, Italy).

#### 2.13.2. MTT Assay

All typologies of samples were irradiated with UV light for sterilization, rinsed with PBS 1X, and incubated in DMEM for 24 h and 48 h at 37 °C, 5% CO_2_. NHDFs were seeded in a 96-well plate at a concentration of 5 × 10^3^ cells/well, and after 24 h of incubation (at 37 °C and 5% CO_2_) cell medium was replaced with sample extracts. After 24 h of cell incubation at 37 °C, 5% CO_2_ samples extracts were replaced with an MTT solution (5 mg/mL in DMEM), and after 2.5 more hours of incubation (37 °C and 5% CO_2_) the MTT solution was analyzed by employing a microplate reader HiPo MPP-96 (Biosan medical technologies, Riga, Latvia) at a selected wavelength of 570 nm. Cells cultured on tissue culture polystyrene (TCPs) without sample, and cells seeded in TCPs and treated with phenol 99.0% *v*/*v* (Sigma Aldrich; Milan, Italy), both treated under the same conditions, were employed as positive and negative controls, respectively.

#### 2.13.3. Morphological Characterization

A morphological characterization was carried out on NHDFs exposed to sample extracts using a light microscope. Before adding MTT solution, cell morphology was observed by employing a LEICA DMIL LED Fluo optical microscope (Leica Microsystems, Milan, Italy), allowing for the detailed observation of any alterations in cell morphology, such as detachment, swelling, or changes in cell density or shape.

### 2.14. Preliminary Analysis of the Life Cycle of Greenhouse Gas (GHG) Emissions

A preliminary analysis of the life cycle of greenhouse gas (GHG) emissions for the developed partially biobased medical textile was conducted using the standardized LCA methodology outlined in the ISO 14040-14044 [29] series by the International Organization for Standardization. The aim was to identify key environmental hotspots in the context of GHG emissions across the three main stages, from tomato peel to the medical device, using the supercritical carbon dioxide (CO_2_) extraction method. This enabled system improvements for enhanced environmental sustainability. The three stages were isolation and purification (supercritical fluid extraction technique) (Stage I), depolymerization and monomer recovery (Stage II), and valorization path (Stage III). The functional unit was a single-use surgical face mask, weighing 454.4 mg of PLAPCL–Cut fibers, serving as the reference flow. The system boundary was set up following a cradle-to-factory gate approach. At this early stage, the study was based on the IPCC 2021 method, which contains the Global Warming Potential (GWP100) climate change factors of IPCC with a timeframe of 100 years. This study was conducted on a pilot scale for Stage II and III, and on a laboratory scale for Stage III, as this last stage has not yet been able to move to upscaling. Therefore, this preliminary study is only the first step, and additional in-depth studies will be performed as the research progresses. Lab scale data are more uncertain than data from larger-scale production, so the results should be interpreted with caution and only as a guide for how to continue improving the process. The primary lab and pilot data were incorporated into the SimaPro LCA software version 9.6, and the Ecoinvent database version 3.10 (allocation cut-off by classification as system model) was used as a background source for the processes used, before evaluating the foreground data. Finaly, a comparison with fossil-based single-use masks was also made to observe the differences [30]. The construction of the fossil-based face mask included an aluminum nose piece, polyurethane, and a composite of melt-blown polypropylene (PP) sandwiched between a layer of spun-bond PP with a specific grammage.

### 2.15. Statistical Analysis

The results are reported as mean ± standard deviation. Statistical analysis of the results was carried out employing a one-way or two-way ANOVA followed by Tukey post hoc test. A *p*-value < 0.05 was considered statistically different.

## 3. Results and Discussion

### 3.1. Solution Characterization

The rheological behavior and surface tension are fundamental parameters in determining the processability of materials via electrospinning. To optimize these parameters, the concentrations of PLAPCL and cutin were systematically adjusted. Drawing on previous studies [31,32], a PLAPCL concentration of 15% *w*/*v* was selected as optimal for subsequent experiments. The electrospinning process involves the uniaxial stretching of a charged jet. If the concentration of the polymeric solution is low, the applied electric field and surface tension cause the jet to break into fragments and the formation of beads or beaded fibers.

By increasing the concentration of the polymeric solution, it is possible to increase its viscosity because of the higher number of polymeric chain entanglements. Sufficiently high solution viscosity will result in the overcome of the surface tension and will ultimately result in uniform electrospun nanofibers without the presence of beads [33]. A graph reporting the variation of PLAPCL solutions viscosity with polymer concentration is reported in Figure S1, and it was confirmed that a polymer concentration of 15% *w*/*v* allowed the formation of macromolecular chains entanglements, which is a fundamental requirement for the formation of a stable Taylor cone and polymeric fibers during electrospinning processing [34].

**Figure 1:**
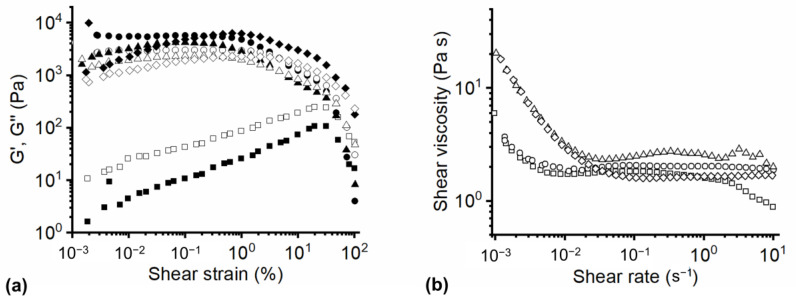
Rheological characterization of the PLAPCL and PLAPCL–Cut solutions: (**a**) elastic (G′, filled symbols) and viscous (G″, empty symbols) moduli vs. Shear strain of PLAPCL solutions ■, and PLAPCL solutions with the addition of 0.75% *w*/*v* ●, 1.5% *w*/*v* ▲, and 2.7% *w*/*v* ♦ of Cut; (**b**) shear viscosity vs. Shear strain of PLAPCL solutions ■, and PLAPCL solutions with the addition of 0.75% *w*/*v* ●, 1.5% *w*/*v* ▲, and 2.7% *w*/*v* ♦ of Cut.

**Figure 2:**
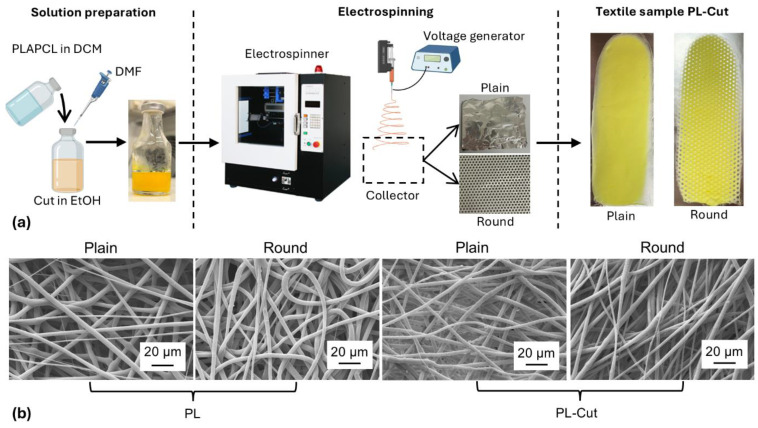
(**a**) Schematic representation of the preparation of the PLAPCL–Cut suspensions, of the electrospinning process, and representative pictures of PL–Cut samples obtained with two different morphologies: Plain and Round; (**b**) SEM analysis performed on the PL and PL–Cut samples fabricated with Plain and Round morphologies.

The amplitude sweep test and shear strain ramp test were carried out on PLAPCL solutions (15% *w*/*v*) containing different amounts of Cut to determine their suitability to be processed through electrospinning. In Figure 1 are reported the graphs obtained from the rheological characterization of the developments PLAPCL and PLAPCL–Cut solutions, and Figure 2a presents a schematic representation of PLAPCL–Cut suspension preparation.

In Figure 1a are reporting the elastic (G′) and the viscous (G″) moduli of the developed suspension obtained through the amplitude sweep test. PLAPCL solutions showed higher loss modulus than storage modulus at every tested shear strain.

The addition of Cut determined an increase of both G′ and G″ values, and it also determined a different rheological behavior of the suspensions. PLAPCL–Cut5 and PLAPCL–Cut10 showed a higher elastic modulus than viscous modulus at low shear strain values, but the two graphs crossed between 1 and 10% and the viscous modulus became the dominant constituent. This phenomenon is usually associated with the breakdown of the inter-macromolecular interactions and the transformation of the polymeric solution behavior from solid-like to liquid-like [35]. PLAPCL–Cut18 showed higher elastic modules than viscous modulus for almost all tested amplitude values. The two graphs crossed only at the highest tested shear strain value, confirming the dominant elastic behavior of the suspension. This rheological behavior determines the higher stability of the Taylor cone and of the jet, assuring a better solution processability through electrospinning [36].

Figure 1b shows the variation of solution viscosity with shear rate at different Cut content. PLAPCL graphs show three different regions characterized by different rheological behavior. In the first region, at low shear rate values, PLAPCL solutions presented a shear thinning behavior and the viscosity decreased with shear rate. This phenomenon may depend on the intermolecular hydrogen bonds breaking faster than new ones are formed. In the second region, the solutions showed a Newtonian behavior, and the viscosity did not change when increasing the shear rate. In the last region, solution viscosity again decreased with the shear rate. Adding 0.75% *w*/*v* of Cut to the PLAPCL solution did not change its rheological behavior in the first two regions, but it determined the disappearing of the third region. Indeed, PLAPCL–Cut5 (Table 1) solutions showed Newtonian behavior from 0.01 s^−1^ to 10 s^−1^. Increasing Cut content to 10 and 18% *w*/*w* determined an increase in solution viscosity in the first region of up to 20 Pas; this might depend on the increase of Cut particles suspended in PLAPCL solution and on the increase of interactions between the polymer and Cut. At the shear rate corresponding to the deformation rate felt in the needle (10 s^−1^ [37]), Cut-based suspensions showed increased viscosity in respect to PLAPCL solution and the absence of shear thinning behavior at high shear rates, implying that inter-macromolecular interactions and interactions between PLAPCL and Cut are formed at the same rate at which they are broken, even at high shear rate. This last condition is necessary for the formation of the Taylor cone and has a stable jet and determined the suitability of Cut-based suspensions to be processed by electrospinning and even increase the processability of the PLAPCL. Therefore, the PLAPCL–Cut18 suspension resulted to be the best one among all the suspensions studied to be processed by electrospinning, and it was selected to obtain the final textile.

Surface tension represents another important property to determine solution spinnability. PLAPCL–Cut18 solutions presented a surface tension of 45.5 mN/m, resulting in suitable electrospinning [27].

### 3.2. Electrospinning of PLAPCL and PLAPCL–Cut Solutions

Electrospinning fabrication parameters were optimized for the processing of PLAPCL and PLAPCL–Cut18 and the development of a non-woven textile. To the best of the authors’ knowledge, this is the first time that Cut is blended with PLAPCL and processed through electrospinning. The electrospinning technique was selected to develop the devices because it allows the formation of microfibers with highly reproducible diameters, ranging from 2 nm to several micrometers [38]. Moreover, differently from other conventionally employed techniques, involving the melting and extrusion of the material (e.g., melt blown), electrospinning allows the processing of polymeric solutions, avoiding the high temperatures required for melt processing, thus eliminating the thermal degradation of the materials due to thermo-mechanical stresses [39,40].

The effect of voltage variation on sample fabrication was studied on PLAPCL solutions; the applied voltage varied between 22 and 28 kV. A voltage of 22 kV was too low to completely deform the liquid droplet into the Taylor cone, so a solution jet was formed together with solution droplets that fell on the non-woven textile. Increasing the voltage values to 23 kV determined the reduction of the droplet’s dimension. Increasing it to 28 kV also determined the instability of the solution jet, which was deposited in an irregular way on the collector. The obtained samples presented a homogeneous distribution of fibers and were constituted of thick areas with a high number of fibers deposited alternately, with very thin areas with almost no fibers deposited. A voltage value of 25 kV represented the best value for sample fabrication, allowing the homogeneous deposition of the fibers on the collector with no defects. A schematic representation of the solution preparation and the electrospinning process and representative pictures of PL–Cut samples obtained with different morphologies are depicted in Figure 2a.

Two different collectors were employed, one constituted of a smooth aluminum plate, the other constituted by a porous aluminum plate. By using the two collectors, two different typologies of textile were fabricated, the first one characterized by a dense fibrous structure, named Plain, the other characterized by the presence of a homogeneous distribution of round pores, as reported in Figure 2a, named Round. The two different morphologies were intended for textile application into different medical fields. The Plain textile was meant for dressing and filtration purposes for the development of devices such as face masks or surgical gowns. The Round textile was meant for the development of patches for wound healing since the texture allows close contact between the dressing and the wound [27]. The dimensional characterization of the fabricated samples is reported in Table 2.

Dimensional characterization revealed that sample dimensions were not affected by morphology (Plain or Round), but they can be affected by the selected material. In particular, Cut addition determined a significant increase of both Plain and Round sample width, but it also reduced Plain sample thickness. Surprisingly, after the same spinning time, Cut addition did not significantly affect the weight of the sample.

### 3.3. Morphological Characterization of the Developed Patches

Scanning electron microscopy (SEM) was carried out on the electrospun PL and PL–Cut samples obtained with Plain and Round morphologies to assess the effective formation of fibers, to measure their diameter, and to determine the absence of fabrication defects. In Figure 2b, the obtained micrographs are reported. The micrographs confirmed that the selected fabrication parameters allowed the formation of fibers with no structural defects, such as beads. From the micrographs, it was also possible to see that fibers of all samples are characterized by a smooth surface, indicating that Cut particles are not exposed on the surface but are encapsulated in the core of the fibers. This phenomenon has affected the surface properties of Cut-containing samples.

SEM micrographs also allowed the determination of the mean fiber diameters that are reported in Table 1. Samples fiber diameter ranged between 3 and 4 μm, with no significant differences among different samples. This confirmed that diameter was not affected either by the different morphology nor by the presence of Cut, and this may also be an indication of the intimate mixing of the two materials into the fibers, confirming the good compatibility between PLAPCL and Cut. Fiber diameters as well as their random orientation are suitable for the employment of the developed devices for the fabrication of surgical face masks, considering that commercially available surgical face masks made of polypropylene usually present filtration layers composed of randomly oriented fibers with mean diameter ranging from 1 to 10 μm [41,42]. Fiber diameters and random orientation are also suitable for the application of the obtained devices for the development of dressings for wound healing [27].

### 3.4. Cutin Encapsulation Efficiency

**Figure 3:**
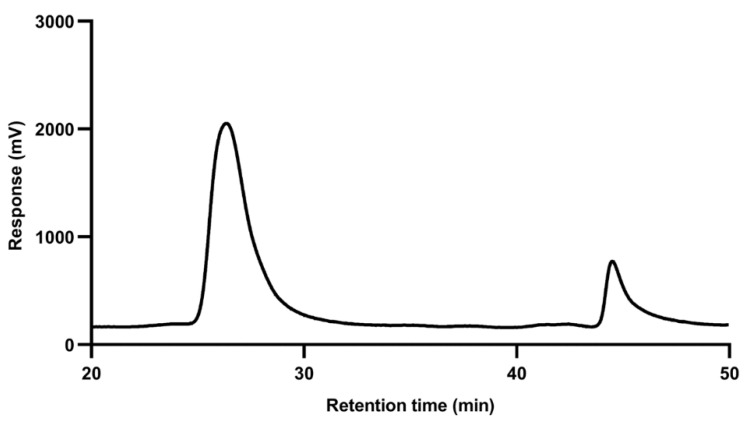
Representative GPC chromatogram of PL–Cut.

For the determination of Cut encapsulation efficiency, an exact weighted amount of PL–Cut textile was dissolved in THF and was analyzed through GPC. The chromatogram showed two signals, the first one with a retention time of about 26 min was associated with PLAPCL presenting higher molecular weight than Cut, and the second one with a retention time of about 45 min was associated with Cut. In Figure 3, a representative chromatogram of a PL–Cut sample is reported. To determine the amount of Cut encapsulated in the dissolved sample, the height of the Cut signal was compared with a previously developed calibration curve, obtained by analyzing Cut solutions at different concentrations to determine Cut concentration. Cut encapsulation efficiency was calculated according to the following equation (Equation (1)):

Three measurements were carried out on three different samples, and the encapsulation efficiency resulted to be 97.4 ± 1.2%, which is consistent with the technique employed for the samples fabrication.

### 3.5. Thermal Characterization

**Figure 4:**
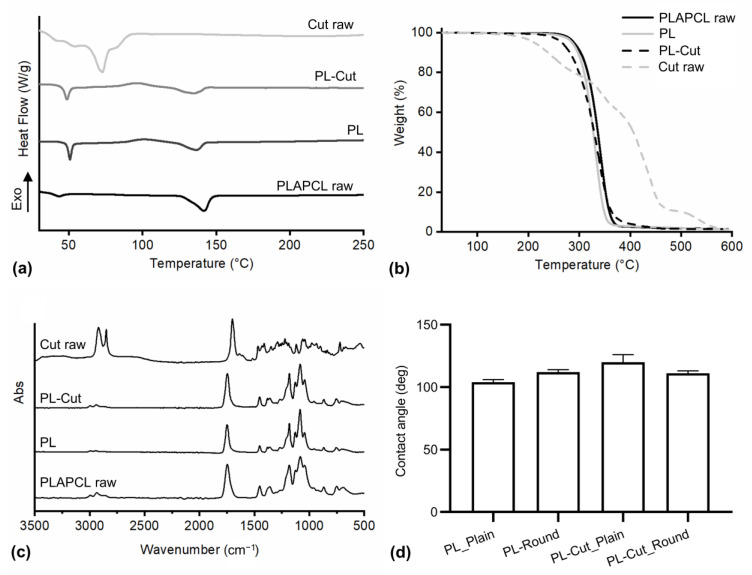
Thermal characterization and FT-IR analysis of PLAPCL raw, PL, PL–Cut, and Cut raw (**a**) DSC thermogram; (**b**) TGA thermogram; (**c**) FT-IR spectra; (**d**) contact angle values of textiles constituted of different material (PL and PL–Cut) and different morphologies (Plain and Round); data are reported as mean ± standard deviation (*n* = 3).

Thermal characterization was carried out through differential scanning calorimetry (DSC) and through thermogravimetric analysis (TGA). In Figure 4a are depicted the DSC thermograms obtained by analyzing the raw polymer (PLAPCL raw) for the PLAPCL electrospun sample (PL), for the PLAPCL–Cut electrospun sample (PL–Cut), and for the raw cutin (Cut).

The DSC thermogram of PLAPCL raw showed two endothermic peaks related to the melting of the polymer crystals. The first peak was associated with the melting of PCL crystals, showing a melting temperature (T_m1_) of 43 °C, while the second one was associated with the melting of the PLA crystals, showing a T_m2_ of 142 °C. The melting enthalpies allowed for the calculation of the crystallinity degree (χ) of PCL and PLA blocks, resulting respectively in 2.3% and 30.5%. The thermograms of Cut raw showed different superimposed melting phenomena (the most intense of which showing a T_m_ of 72 °C) related to the presence of different molecules constituting Cut raw (e.g., 10,16-dihydroxyhexadecanoic acid, and 10,18-dihydroxyoctadecanoic acid) [43].

The DSC thermograms of PL showed a first endothermic peak associated, as in the previous case, with the melting of PCL block crystals (T_m1_: 51 °C and χ: 6.3%), an exothermic peak related to the cold crystallization of PLA blocks happening at a temperature (T_cc_) of 102 °C, and the final melting of the PLA blocks crystals (T_m2_: 137 °C χ: 15%). The addition of Cut determined a reduction of T_m1_ to 49 °C, T_cc_ to 96 °C, and T_m2_ to 135 °C, without affecting χ values. This reduction phenomenon may be ascribed to the presence of Cut molecules creating defects in the polymeric crystal latex, allowing their melting at lower temperatures. No signals related to Cut were visible in the PL–Cut samples, probably because the Cut crystals were molten during solution preparation and were not able to crystallize again with them being dispersed among the macromolecular chains.

TGA analysis (Figure 4b) allowed the evaluation of the device’s thermal stability. The thermograms of PLAPCL raw as well as PL showed a single weight loss phenomenon due to the polymer thermal degradation into volatile products. The thermogram of Cut showed different superimposed degradation phenomena. Similarly to PLAPCL raw and PL, PL–Cut showed a single degradation phenomenon caused by the overlapping of the degradation of the polymer and Cut. Degradation temperatures (T_deg_) of the different samples were determined as the onset temperatures, and it resulted that PLAPCL raw showed the highest T_deg_ 319 °C, followed by PL (T_deg_ of 314 °C). Cut raw showed a T_deg_ of 200 °C. PL–Cut showed a T_deg_ of 307 °C, and together with the absence of a second degradation step it indicated an increase in Cut stability.

### 3.6. Infrared Spectroscopy

FT-IR analysis was carried out on PLAPCL raw, Cut raw, PL, and PL–Cut, and the obtained spectra are reported in Figure 4c. The spectra of PLAPCL raw showed typical signals of polyesters: three signals at 3000, 2950, and 2900 cm^−1^ associated to the stretching of the CH_3_, CH_2_, and CH of lactic acid units and CH_2_ of caprolactone units, and a signal at 1750 cm^−1^ related to the stretching of the carbonyl groups of esters and a band ranging from 970 to 1280 cm^−1^ related to the bending of the ester bonds [44]. The spectrum of Cut raw showed two absorption bands at 2920 cm^−1^ and 2850 cm^−1^ related to, respectively, the asymmetric and symmetric stretching of the methylene groups. The absorption band at 1705 cm^−1^ is associated with the stretching of the carbonyl groups of carboxylic acid groups involved in hydrogen bonds and in the formation of dimeric structures [45]. The formation of dimeric structures is also confirmed by the absence of the absorption bands of the hydroxyl groups usually found (-OH) at 3400–3500 cm^−1^ [46]. PL and PL–Cut spectra are like the spectrum of PLAPCL raw; therefore, Cut did not affect the functional groups of the polymer. It was not possible to detect signals related to Cut in the PL–Cut spectrum, probably because ATR FT-IR analysis is a surface analysis and most of Cut is encapsulated in the core of the fibers.

### 3.7. Determination of the Wettability of the Textiles

Contact angle measurement was carried out on PL and PL–Cut samples, presenting plain morphology and a round morphology to determine their wettability.

In Figure 4d are reported the contact angle values. As it is possible to see from the graphs, all samples presented contact angle values higher than 90°, confirming the hydrophobicity of the developed textiles. The hydrophobicity of the textile is determined by the hydrophobic material (considering that both PLA and PCL constituting the copolymer are hydrophobic materials [47,48]) and increased by the presence of pores [49]. The morphology did not affect contact angle values, probably because the droplet is too small, and it is completely deposited on the dense part without being affected by the round pores. Adding Cut to the device seems to increase the contact angle values of the samples, but the difference is not significant. This result further confirm that Cut is dispersed in the core of the fibers and only a small part is exposed on the surface. The hydrophobicity of the devices further confirmed their suitability to be applied for the development of surgical face masks (usually constituted of non-woven polypropylene layers showing a contact angle of 101° [50]) because it avoids the surface getting wet when encountering saliva and other physiological fluids and avoids their absorption [51]. Contact angle values are also suitable for device application for wound healing. They are like the contact angle values of Mepilex Lite (~90 °), a foam dressing marketed by Molnlycke and designed for the treatment of acute and chronic wounds with low exudation [27].

### 3.8. Stability After γ-Irradiation

The sterilization of the developed devices was performed by employing γ-irradiation as the sterilization technique, it being one of the most employed by biomedical industries. The stability of the material constituting the devices was evaluated through GPC analysis, which allowed the determination of the molecular weight of PLAPCL and Cut before and after sterilization. As stated above, the chromatogram of a PL–Cut sample presented two different signals, the first one with a retention time of about 26 min, associated with PLAPCL, and a second one with a retention time of about 45 min, associated with Cut.

**Figure 5:**
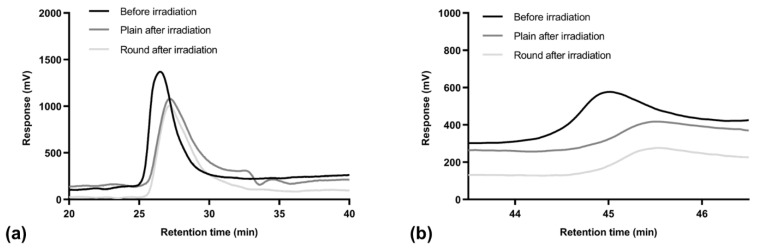
GPC chromatograms of the PL–Cut textile before and after γ-irradiation: (**a**) signal related to PLAPCL; (**b**) signal related to Cut.

In Figure 5 are depicted the signals of the PLAPCL (a) and Cut (b) through the GPC analysis of both morphologies of PL–Cut samples.

The chromatograms in Figure 5 show that both signals presented an increased retention time after γ-irradiation, which indicates a reduction in the molecular weight of PLAPCL and Cut due to degradation phenomena. The number average molecular weight of PLAPCL before γ-irradiation was 131 kDa, and it decreased to 79 and 93 kDa for the Plain and the Round samples, respectively. The number average molecular weight of Cut before γ-irradiation was 537 Da, and it decreased to 466 Da and 471 Da for the Plain and the Round samples, respectively. Upon γ-irradiation, PLAPCL reduced its molecular weight by 40% for the Plain sample and by 30% for the Round sample, while Cut reduced its molecular weight by 13% and 12%. By considering these data, it can be hypothesized that Cut is more stable to γ-irradiation, and its blending to the device material can be a useful way to increase device stability to γ-sterilization process.

### 3.9. Mechanical Characterization

**Figure 6:**
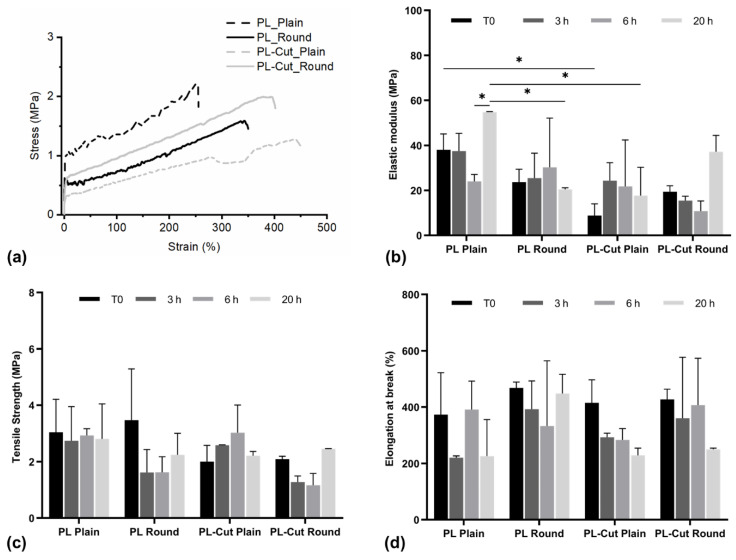
Mechanical characterization: (**a**) representative stress–strain curves of PL and PL–Cut samples; (**b**) elastic modulus, (**c**) tensile strength, and (**d**) elongation at break of the different samples after fabrication (T0) and at selected times of incubation in distilled water; all data are reported as mean + standard deviation (*n* = 5); * values marked by the symbol are statistically different (*p* < 0.05).

Mechanical characterization of the textile was carried out through tensile test. The test was performed after fabrication (T0) and after incubation in distilled water for 3 h, 6 h, and 20 h. The results are reported in Figure 6.

The resulting stress–strain curves are constituted by three different regions. In the first region, the stress increases linearly with the strain; this region is called the elastic region and depends on the deformation resistance of fibers [52]. The second region is characterized by a further increase in stress with strain but with a lower rate. This phenomenon is usually associated with the unfolding of the chain entanglement and the sliding of the fibers [52]. The last region represents the breaking of the fibers and of the sample, and it is characterized by the rapid decrease of stress with the strain.

From the stress–strain graphs, data relative to the elastic modulus, to the tensile strength, and to the elongation at break of the samples characterized by different morphologies (plain and round) and constituted of different materials (PL and PL–Cut) were obtained.

From the comparison of the mechanical properties of the samples tested at T0, it resulted that Cut blending reduced the Plain sample’s elastic modulus, but it did not reduce the elastic modulus of the Round samples. Sample incubation in distilled water did not significantly affect the samples’ mechanical properties, except for the PL Plain samples, which presented an increased elastic modulus after 20 h of incubation in comparison with the PL Round samples and PL–Cut Plain samples. In general, the results of the mechanical characterization agrees with data reported in the literature stating that PLA and PCL complete degradation in physiological environment requires 2–3 years [53,54]. This feature may be interesting in evaluating the reusability of the developed devices, to further increase their environmental sustainability.

In general, sample elastic modulus values ranging between 20 and 40 MPa and tensile strength ranging between 1.8 and 3.2 MPa confirmed the suitability of the developed textile to be employed as the middle layer of a surgical face mask (elastic modulus ~30 MPa, and tensile strength 4 MPa [55]), even after coming in contact with aqueous fluids for 20 h; however, elongation at break is significantly superior to that of the conventionally used surgical face mask middle layer [55]. The developed textiles also presented suitable mechanical properties for application for wound healing, showing elastic modulus and tensile strength similar to Acticoat^®^ (elastic modulus ~35 MPa, tensile strength ~4.5 MPa [56]), which is a commercially available wound dressing with antibacterial properties, but with significantly higher elongation at break [56].

### 3.10. Determination of Cytotoxicity

**Figure 7:**
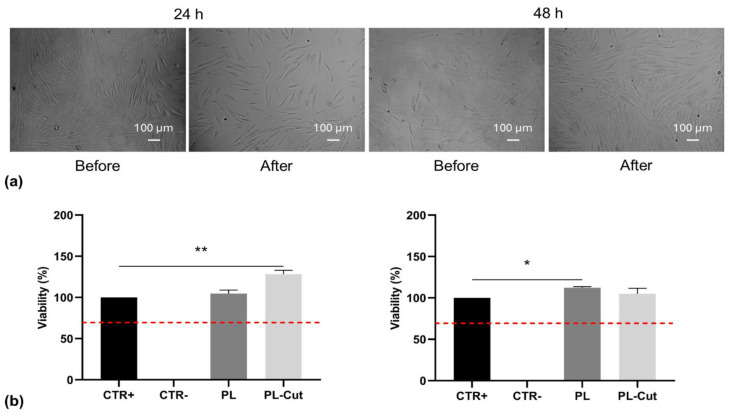
Determination of textile cytocompatibility (**a**) images obtained with optical microscope of NHDF incubated with different PL–Cut extracts; (**b**) MTT assay performed after 24 (**left**) and 48 (**right**) h of incubation; values are reported as mean ± standard deviation (*n* = 3), * marked values are significantly different (*p* < 0.05), ** marked values are statistically different (*p* < 0.01). Red dashed line indicates 70% of cell viability.

The cytotoxicity of the developed devices was evaluated through an indirect MTT assay carried out by employing NHDFs according to ISO10993-09 [57]. Cells were incubated in device extracts, and their viability was evaluated through optical microscopy analysis and through the MTT assay. Both tests were carried out after 24 and 48 h of incubation. In Figure 7, the micrographs obtained and the results of the MTT assay are reported.

Cell morphology was considered as a preliminary evaluation of cell viability. Micrographs (Figure 7a) showed that cells presented long cytoplasmic strands at all the analyzed locations, confirming their viability with just a few round dead cells [36]. These preliminary results were confirmed by the MTT assay (Figure 7b), which was carried out after 24 and 48 h of incubation, as requested by the ISO standard [57]. At both the selected times, cell viability values are higher than 70%, which, according to the standard, represents a threshold value determining the cytocompatibility of the developed devices. This test confirmed that the toxic organic solvents employed for the preparation of the starting polymeric solutions were effectively removed from the final sample during the fabrication process and they did not affect their cytocompatibility.

### 3.11. Assessing Global GHG Emissions

A preliminary analysis of the life cycle of greenhouse gas emissions was conducted as an early assessment of the PLAPCL–Cut fibers (1 surgical mask), focusing on the emissions associated with its three stages. The results are shown in Table 3. The total CO_2_-equivalent GHG emissions for the medical device were estimated at 0.315 kg of CO_2_ eq per 454.4 mg of PLAPCL–Cut fibers, with 93% of these emissions related to Stage III, where the main contributor is the electricity used to produce cutin-based textile followed by PLAPCL monomer. In Stage I, the use of the green solvent accounts for only 2% of the total impact, followed by Stage II with 5%. Despite the lower percentage for Stage II compared to Stage III, the use of methanol, dichloromethane, and the incineration treatment of these solvents indicate that further improvements are necessary due to the presence of environmentally harmful chemicals. Moreover, Stage III will requires further research to incorporate biobased PLA alternatives. Currently, PLA in medical device products can only be fossil-based due to specific regulations requiring PLAPCL to be fossil-based in medical devices.

Amos Wei Lun Lee’s work [30] analyzed the GHG emissions for a fossil-based face mask, showing results of 0.580 kg CO_2_-eq per 22 g of a single-use surgical face mask (0.026 kg CO_2_-eq g^−1^), while our study presents 0.69 kg CO_2_-eq g^−1^. This demonstrates the importance of reducing electricity use in Stage III, as well as minimizing the use of non-environmentally friendly chemicals in Stage II.

This was the first work to investigate the LCA of the production of partially biobased masks. Other works mainly focused on the performances of biobased masks, but determined their environmental impact by considering the production of biobased fibers [24].

## 4. Conclusions

In this work, a novel method for the fabrication of new non-woven textiles made of a blend based on PLAPCL and agro-food, waste-derived cutin was developed by employing the electrospinning technique. The developed textiles were found to be suitable for medical applications such as the filtering layers of surgical face masks and patches for wound healing. In particular, a Cut/PLAPCL ratio of 0.18 *w*/*w* in the starting suspensions resulted in the optimal composition based on rheological properties and surface tension. Electrospinning parameters were optimized to obtain different textiles based on PLAPCL and the PLAPCL–Cut blend and showed different morphologies: a conventional plain morphology and a round porous morphology. The samples were shown to be constituted by microfibers with no structural defects, and it was demonstrated that electrospinning processing did not degrade PLAPCL and Cut. The suitability of the developed samples for medical applications such as the filtering layer of face masks and patches for wound healing was demonstrated through determination of their hydrophobicity, flexibility, mechanical property stability after incubation in water, and through absence of any cytotoxic effect. Further work will be carried out to increase cutin content in the blend, to reduce devices degradation during sterilization through γ-irradiation, and to reduce GHG emissions, mainly derived from the organic solvents employed for Cut depolymerization and purification, and from the electricity used during devices fabrication.

## Figures and Tables

**Table 1:** Optimized parameters employed for the preparation of PLAPCL solutions and of PLAPCL–Cut suspensions at different Cut concentrations.

Solution ^a^	PLAPCL (% *w*/*v*)	Cut (% *w*/*v*)	Cut/PLAPCL (*w*/*w*)
PLAPCL	15.0	-	0.00
PLAPCL–Cut5	15.0	0.75	0.05
PLAPCL–Cut10	15.0	1.50	0.10
PLAPCL–Cut18	15.0	2.70	0.18

^a^ Solvent system is constituted of DCM: 94.1% *v*/*v*, EtOH: 3.3% *v*/*v*, and DMF 2.6% *v*/*v*. Different PLAPCL concentrations were studied, ranging from 0.1% *w*/*v* to 15% *w*/*v* as well as different Cut concentration of 0.75, 1.50, and 2.70% *w*/*v*.

**Table 2:** Dimensional characterization of the developed textile samples: PLAPCL electrospun textile (PL) and PLAPCL and Cut electrospun textile (PL–Cut), both obtained after 10 min of spinning. Values are reported as mean ± standard deviation (*n* = 5, for fibers diameter *n* = 25).

	Length (mm)	Width (mm)	Thickness (mm)	Weight (mg)	Fiber’s Diameter (μm)
PL Plain	96.3 ± 7.2	22.5 ± 6.2 *	0.24 ± 0.10 *	76 ± 7.0	3.8 ± 1.4
PL Round	93.9 ± 3.7	26.1 ± 5.0 ^§^	0.21 ± 0.07	72 ± 14	3.0 ± 0.8
PL–Cut Plain	99.6 ± 3.6	33.8 ± 4.4 *	0.11 ± 0.02 *	77 ± 5.0	3.3 ± 0.6
PL–Cut Round	102.6 ± 7.0	37.0 ± 4.2 ^§^	0.14 ± 0.02	91 ± 16	3.1 ± 0.9

*, ^§^ values marked by the same symbols are statistically different (*p* < 0.05).

**Table 3:** The total GHG emissions for 454.4 mg of PLAPCL–Cut fibers (1 surgical mask) divided into isolation + purification (supercritical fluid extraction technique) (Stages I), depolimerization + monomer recovery (Stage II), and valorization path (Stage III).

GHG Emissions	Total kg CO_2_eq	0.315
	Total phase 3	0.299
Phase 3	PLAPCL	0.00324
	DCM	1.19 × 10^−5^
	EtOH	9.98 × 10^−8^
	DMF	2.11 × 10^−7^
	Electricity	0.289
	Waste solvent mixture	0.007
	Total phase 2	0.0169
Phase 2	MeOH	0.001
	NaOH	4.90 × 10^−5^
	H_2_O	8.77 × 10^−7^
	HCl 37%	1.30 × 10^−5^
	DCM	0.002
	Municipal waste treatment	2.13 × 10^−6^
	Electricity	7.94 × 10^−6^
	Biowaste, treatment	3.87 × 10^−6^
	Solvent mixture incineration treatment	0.008
	Total phase 1	0.0054
Phase 1	Dry peel tomato	2.25 × 10^−5^
	H_2_O	9.60 × 10^−7^
	CO_2_ gas (green solvent)	0.00145
	Electricity	0.00196
	Waste H_2_O	1.04 × 10^−6^
	Emissions CO_2_ gas (green solvent)	0.00194

## Data Availability

The original contributions presented in this study are included in the article/Supplementary Materials. Further inquiries can be directed to the corresponding author(s).

## Supplementary Materials

The following supporting information can be downloaded at: https://www.mdpi.com/article/10.3390/polym17060810/s1, Figure S1: Specific viscosity variation with PLAPCL concentration, data are reported as mean (*n* = 3).
