# Supplementary material for: Bioengineering the Future: Tomato Peel Cutin as a Resource for Medical Textiles
Source: Polymers (Basel). 2025 Mar 19;17(6):810. doi: 10.3390/polym17060810 (PMC11945004; doi:10.3390/polym17060810)
Supplement: Supplementary file 1 [file polymers-17-00810-s001.zip › polymers-3511418-supplementary.pdf]

Supporting information

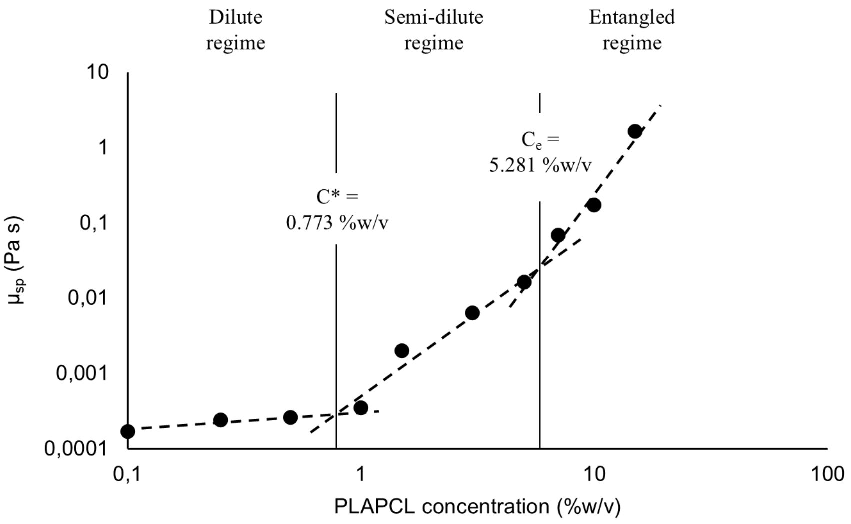

Figure S1 Specific viscosity variation with PLAPCL concentration, data are reported as mean (n=3)
